# Behavior Change App for Self-management of Gestational Diabetes: Design and Evaluation of Desirable Features

**DOI:** 10.2196/36987

**Published:** 2022-10-12

**Authors:** Mikko Kytö, Saila Koivusalo, Antti Ruonala, Lisbeth Strömberg, Heli Tuomonen, Seppo Heinonen, Giulio Jacucci

**Affiliations:** 1 Helsinki University Hospital IT Management Helsinki University Hospital Helsinki Finland; 2 Department of Computer Science University of Helsinki Helsinki Finland; 3 Department of Gynecology and Obstetrics Turku University Hospital Turku Finland; 4 Department of Gynecology and Obstetrics University of Turku Turku Finland; 5 Department of Gynecology and Obstetrics Helsinki University Hospital Helsinki Finland; 6 Department of Gynecology and Obstetrics University of Helsinki Helsinki Finland

**Keywords:** gestational diabetes, mobile app, features, behavior change, digital health, eHealth, telehealth, self-tracking, self-management, personalized health care

## Abstract

**Background:**

Gestational diabetes (GDM) has considerable and increasing health effects as it raises both the mother’s and the offspring’s risk for short- and long-term health problems. GDM can usually be treated with a healthier lifestyle, such as appropriate dietary modifications and sufficient physical activity. Although telemedicine interventions providing weekly or more frequent feedback from health care professionals have shown the potential to improve glycemic control among women with GDM, apps without extensive input from health care professionals are limited and have not been shown to be effective. Different features in personalization and support have been proposed to increase the efficacy of GDM apps, but the knowledge of how these features should be designed is lacking.

**Objective:**

The aim of this study is to investigate how GDM apps should be designed, considering the desirable features based on the previous literature.

**Methods:**

We designed an interactive GDM prototype app that provided example implementations of desirable features, such as providing automatic and personalized suggestions and social support through the app. Women with GDM explored the prototype and provided feedback in semistructured interviews.

**Results:**

We identified that (1) self-tracking data in GDM apps should be extended with written feedback, (2) habits and goals should be highly customizable to be useful, (3) the app should have different functions to provide social support, and (4) health care professionals should be notified through the app if something unusual occurs. In addition, we found 2 additional themes. First, basic functionalities that are fast to learn by women with GDM who have recently received the diagnosis should be provided, but there should also be deeper features to maintain interest for women with GDM at a later stage of pregnancy. Second, as women with GDM may have feelings of guilt, the app should have a tolerance for and a supporting approach to unfavorable behavior.

**Conclusions:**

The feedback on the GDM prototype app supported the need for desirable features and provided new insights into how these features should be incorporated into GDM apps. We expect that following the proposed designs and feedback will increase the efficacy of GDM self-management apps.

**Trial Registration:**

ClinicalTrials.gov NCT03941652; https://clinicaltrials.gov/ct2/show/NCT03941652

## Introduction

Approximately 14% of pregnant women are diagnosed with gestational diabetes mellitus (GDM) worldwide, with the highest prevalence in the Middle East and North Africa (27.6%) and in Southeast Asia (20.8%) and the lowest in Europe (7.8%) and North America and the Caribbean (7.1%) [1]. These recent prevalence findings are consistent with the high prevalence of overall diabetes in these regions, and it has been suggested that the same lifestyle risk factors that underly the overall diabetes, such as obesity and metabolic syndrome, also contribute to an increased risk of GDM. GDM is associated with a range of adverse short- and long-term consequences for both mother and child [2-4]. Although GDM is a temporary condition that lasts until the birth of the child, GDM increases the later risk of type 2 diabetes [5]. ﻿The primary treatment for GDM is through adjustments toward a heathier lifestyle, especially changing one’s diet and increasing exercise [6,7]. It is critical that women with GDM be supported in this behavior change [8]. Although a large body of previous studies on supporting type 1 diabetes and type 2 diabetes self-management with apps exists (eg, [9]), these studies do not consider the design implications for diabetes self-management arising from temporality and pregnancy.

Telehealth interventions that provide frequent feedback from health care personnel on lifestyle and glucose levels have been shown to be effective in improving glycemic control among women with GDM [10]. However, providing this feedback to each woman with GDM by health care professionals daily requires a lot of human work and is costly. GDM management apps with less input from health care professionals are limited and have not been shown to be effective [11-13]. The knowledge on user experience with GDM apps is also limited, although recent studies have provided relevant findings [14-18]. According to a user study by Skar et al [17], the most important features of the GDM app researched were the overview of blood glucose levels, real-time feedback from fingertip measurements, and information about nutrition. However, their app did not provide any automatic feedback or social support, which Kytö et al [14] argued was among the desirable features of mobile apps for GDM self-management. Other important features of GDM self-management apps are related to competence to manage GDM, personalization, reliable information, support for dual processing (ie, habitual and sense-making reasoning), and integration to existing health care services [14].

Although the previous studies on desirable features [14] have considered *what* the GDM apps for behavior change should cover, *how* to design these features has remained largely open and feedback from women with GDM has not been considered. Therefore, the aim of this study is to gather the experience of women with GDM with implementations of the desirable features in a GDM app prototype.

## Methods

### Research Design

To investigate how the desirable features of GDM apps [14] should be incorporated, our research design consisted of 2 phases: First, we designed a GDM prototype app consisting of implementations of the features [14], and second, we evaluated the experiences of these features with semistructured interviews. The prototype was targeted toward women with GDM who manage their diabetes with lifestyle adjustments and do not require medication (eg, metformin or insulin). As we designed a prototype whose purpose was to gather experiences from a large scale of features, it was a horizontal, experience prototype [19]. Our focus was on the content of the different features, and their functionalities were not implemented in detail (ie, all the buttons and actions did not function).

The purpose of the interviews was to investigate how the women with GDM perceived the example implementations of the desirable features and how they perceived the app for supporting the management of GDM in general. Participants (n=10 women with GDM) were recruited from maternity and antenatal clinics in the Helsinki Metropolitan Area (Finland).

### Ethical Approval

The study was performed in compliance with the Declaration of Helsinki and approved by the Ethics Committee of the Helsinki University Hospital (HUS-2165-2018-3).

### GDM Prototype App

#### Design Rationale for the Prototype

The design of the prototype was based on previous work by Kytö et al [14], which provided initial suggestions on how desirable features could be designed (summarized in Table 1). We had a multidisciplinary research group consisting of health care personnel who are experienced in treating women with GDM (a gynecologist, midwives, and diabetes nurses), a social psychologist, a user researcher, and an interaction designer to brainstorm the implementations of these features in workshops. The final layout of the prototype was designed by an interaction designer.

**Table 1:** Desirable features of mobile apps supporting behavior change in management of GDM^a^, as described by Kytö et al [14].

Feature	Detail
1. Increase competence to manage GDM with automatic feedback and interactive exploration.	This feature highlights the need for feedback on lifestyle and data exploration in the learning process to manage GDM. In apps, women with GDM have so far received feedback from health care professionals [10], and the perception and effectiveness of automated feedback are largely unknown [14]. Lentferink et al [20] showed that praise and suggestions are especially useful behavior change techniques in mobile apps supporting a healthier lifestyle.
2. Increase autonomy by enabling personalization.	The ways to manage GDM are highly individual and significantly vary from person to person [8]. Personalization has a great impact on the perceived effectiveness of telehealth care in women with GDM. However, it has so far been limited to personalization of the app but not to treatment suggestions [14].
3. Provide social support, especially from the partner.	Encouragement by people with close relationships is important for women with GDM [8,21-24]. The partner’s support is seen as especially valuable in effecting a behavioral change in women with GDM, such as increasing exercise [25], but it has not been considered in existing GDM apps [14]. To support the partner’s willingness to use a GDM app, Peyton et al [26] suggested that pregnancy apps should not be too feminine.
4. Support normal pregnancy and debunk myths about GDM.	Women with GDM want reliable information about their disease [21,27], and this information should be in the same app as information about pregnancy [17].
5. Support dual processing as pregnancy is life changing.	Both reflective and habitual processes should be supported in GDM apps. Especially, habits can be considered important with women diagnosed with GDM, as they are generally well motivated to manage GDM but overwhelmed in their daily life [8]. Motivation for maintaining a healthier lifestyle typically decreases after the birth of the baby [17]. Habits could help maintain a healthier lifestyle after the pregnancy also and thus decrease the probability of type 2 diabetes. Between reflective and habitual processes exists contextual fluid reasoning, which is used in creating simple rules between blood glucose levels and lifestyle [28].
6. Integrate the app with normal pregnancy and existing health care services.	In telehealth solutions for GDM management, tight integration between health professionals and women with GDM has been shown to be important [10]. This plays a significant role in the perceived usefulness of the GDM apps [29,30].

^a^GDM: gestational diabetes mellitus.

#### The Evaluated GDM Prototype App

**Figure 1:**
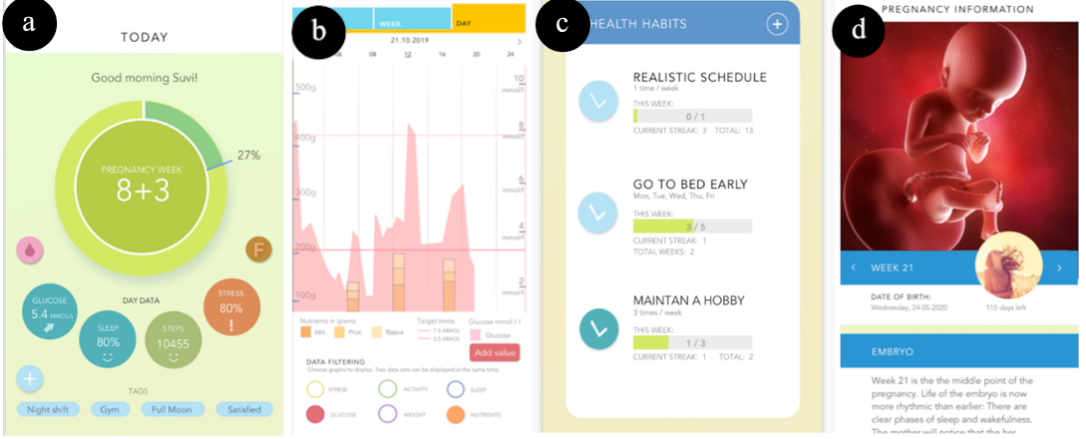
(a) Main page showing pregnancy weeks and recent self-tracking data, (b) data visualization view (bringing self-tracking data into 1 view), (c) habit formation and tracking tool, and (d) general information about pregnancy, the fetus, and GDM. GDM: gestational diabetes mellitus.

**Figure 2:**
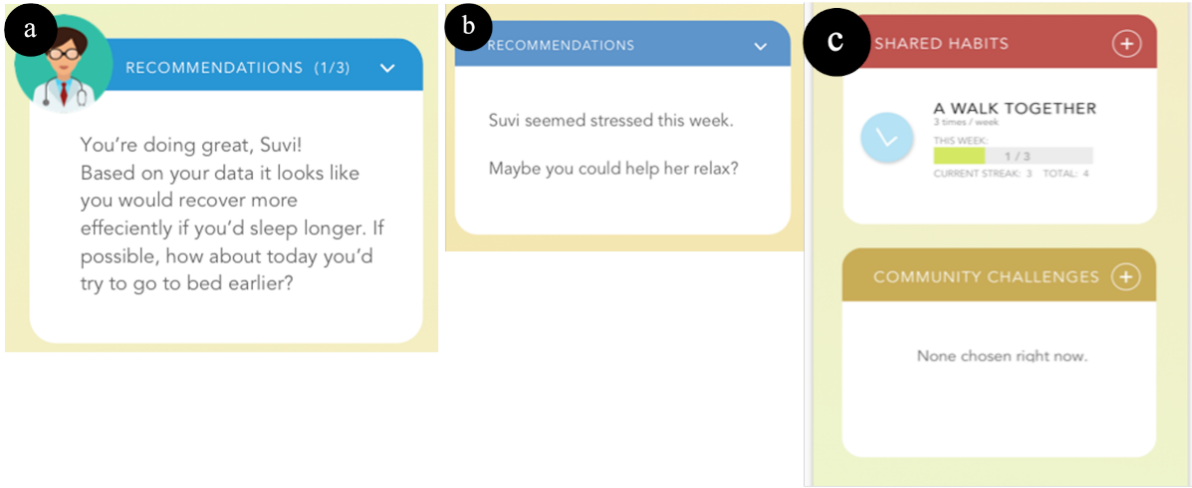
(a) Written reinforcement and feedback, (b) a suggestion for the partner, and (c) shared habits and a community challenge view.

The desirable features [14] were implemented in the prototype app (see Table 2, Figure 1, and Figure 2). The prototype was interactive in nature and ran on a mobile phone’s browser. Users could tap buttons on the screen to navigate pages and scroll. The prototype was implemented using inVision [31], which creates browser-based prototypes. A browser is not the platform for the future actual app, as there will be native iOS and Android versions. In this study, participants were given an iPhone 6, which used the Safari browser to run the prototype.

**Table 2:** Implementations of desirable features [14] in the prototype app.

Feature	Implementation
1. Increase competence to manage GDM^a^ with automatic feedback and interactive exploration.	We investigated this first desirable feature in the form of providing interactive self-tracking data exploration (Figure 1b) and feedback in the form of praise and suggestion (Figure 2a).
2. Increase autonomy by enabling personalization.	We sought to customize the app by providing 2 functionalities, (1) a habit tool (see Figure 1c) and (2) the ability to add a profile picture, name, and expected date of birth into the app.
3. Provide social support, especially from the partner.	We incorporated features to provide social support from both the partner and other women with GDM. For the partner, we designed a view where they could receive suggestions from the app (Figure 2a) and a “Shared habits” functionality that provided the possibility for the partners to do things together (Figure 2b). The content would be provided to the partner through a dedicated partner’s version of the GDM app. For women with GDM, we designed a community challenge where they could support each other to achieve a common goal (Figure 2c).
4. Support normal pregnancy and debunk myths about GDM.	The app provided a draft information section about pregnancy (eg, development of the fetus, as shown in Figure 1d), GDM, nutrition, and physical activity.
5. Support dual processing as pregnancy is life changing.	We supported dual processing by implementing 2 different types of visualizations, an area graph to support reflective thinking (Figure 1b) and the latest values to support habitual thinking (Figure 1a). We also created a habit formation and tracking tool to support more autonomous behavior. The reflective visualization was incorporated by visualizing blood glucose levels, nutrition, and physical activity in a single area graph (Figure 1b). To increase the ecological validity of interpreting data, we visualized actual data recorded from 1 GDM woman before the study. The glucose data (mmol/L) were recorded using Medtronic’s continuous glucose meter Enlite [32], nutrition data (macros in grams) were acquired from a food diary (kept for 3 days) and validated by dietitians, and physical activity data (steps) were recorded with a Garmin Vivosmart 3 activity bracelet. The habitual visualization was provided by showing the most recent values on the first page of the app. A small arrow (Figure 1a) indicating the trend in recent blood glucose levels was added. In addition, to follow customized habits, we designed a view where participants could track their habits (Figure 1c) against goals they had set up.
6. Integrate the app with normal pregnancy and existing health care services.	The app showed the gestational weeks on the entry page (Figure 1a), pregnancy information (Figure 1d), and recommendations by a health care professional (Figure 2a) related to this desirable feature.

^a^GDM: gestational diabetes mellitus.

### Recruitment and Data Collection

Our goal was to recruit 10 women with GDM to the study from maternity and antenatal clinics in the Helsinki Metropolitan Area (Finland). The clinic nurse asked women with GDM at least at 24 gestational weeks about their interest in participation. If interested, the study nurse contacted the mother with more information about the study and confirmed eligibility. Exclusion criteria were type 1 or type 2 diabetes, use of medication that influences glucose metabolism (eg, oral corticosteroids, metformin, insulin), a GDM diagnosis in previous pregnancies, current substance abuse, severe psychiatric disorder, significant difficulty in cooperating (eg, inadequate Finnish language skills), and significant physical disabilities that prevent the use of a smartphone or moving without aid.

Data were collected using the following procedure. After obtaining informed consent, we collected background information (eg, age, pregnancy weeks, and familiarity with mobile apps) through a questionnaire. Next, the research assistant first briefly presented the idea of the prototype and its limitations (it was possible to move between different screens, but the features were not fully implemented) and then instructed the participant to freely browse the prototype (see the Evaluated GDM Prototype App section) and encouraged her to think aloud [33] and voice any possible questions or comments that might come to mind while doing so. This free exploration of design features was chosen as we wanted to gather experiences of the features rather than examining in detail the usability conducting specific tasks with the app. This is a typical approach in user experience studies [34]. If the participant did not find all the screens available in the prototype, the research assistant showed her the screens she had missed. The research assistant asked questions about the features, and after the participant evaluated each feature, the research assistant asked more general questions about the app (see Textbox 1 for the main interview questions). The research assistant also asked follow-up questions when elaboration of the participant’s answer was needed. Regarding desirable features related to social support from the partner (desirable feature 3), we asked participants to evaluate the “femininity” of the app on a scale from 1 (not feminine at all) to 10 (very feminine), as low femininity has been argued to be important for male users [26].

Sessions were conducted in quiet places that were easiest for the participants to travel to and were conducted in their native language. Interviews lasted approximately 1 hour. Interviews were audio-recorded and transcribed. Quotes provided in the results were translated to English verbatim, and filler words, such as “er,” were removed during translation.

**Main interview questions.**

**Main questions about features**

- What do you figure out from this feature? What do you think about it?
- What would you like to have in this feature? How would you develop it?
- Would you need help using this feature?

**Main questions about the app**

- What do you think about the app?
- How do you think the app could help in managing blood glucose?
- How would it feel to use this app in everyday life?
- What do you think about the number of features? Too many or too few? Why?
- What do you think about the look and feel of the app? How feminine do you perceive it to be?

### Analysis

Interviews were transcribed, and 2 researchers familiarized themselves with the interviews by reading the transcripts. The analysis was conducted according to the Framework method, which is a recommended approach for multidisciplinary health research [35]. First, 2 researchers read the interviews and coded them using desirable features (see Table 1) as predefined codes, as they were the focus of the analysis. In cases when content outside these codes emerged, we added new codes. The coding was performed using ATLAS.ti [36]. We ended up with 39 codes and grouped them thematically into larger categories. In the Results section, we present the categories in relation to the desirable features and indicate the frequency of each category.

## Results

### Participants

In total, 10 women with GDM were recruited to the study and interviewed. Table 3 presents their background information. The age of the participants varied from 24 to 40 years (mean 33.6 years, SD 4.4 years), and the gestation weeks varied from 27.1 to 37.0 weeks (mean 33.6, SD 2.7 weeks). Participants were familiar with using various mobile apps, as they agreed with the statement “I am used to using various mobile apps,” with a mean score of 4.3 out of 5 (SD 0.9). The mean scores for the statements “I am used to using physical activity sensors (eg, Fitbit, Vivosmart, and Polar)” and “I am familiar with measuring blood glucose” were 2.4 (SD 1.4) and 4.5 (0.5), respectively.

**Table 3:** Background information about women with GDM^a^ who participated in the study. Regarding the statements, participants responded on a Likert scale (1=strongly disagree to 5= strongly agree).

Participant ID	Age (years)	Weeks of gestation, n	Likert score for “I am used to using various mobile apps.”	Likert score for “I am used to using physical activity sensors (eg, Fitbit, Vivosmart, and Polar).”	Likert score for “I am familiar with measuring blood glucose.”
1	36	35.0	4	3	5
2	32	33.3	4	2	4
3	40	31.2	4	2	4
4	24	33.7	5	2	5
5	31	35.6	4	2	4
6	31	30.3	5	1	4
7	32	36.6	2	1	5
8	36	37.0	5	5	5
9	35	34.8	5	1	4
10	39	28.1	5	5	5

^a^GDM: gestational diabetes mellitus.

### Increased Competence to Manage GDM With Automatic Feedback and Interactive Exploration

Most of the participants (9/10, 90%) wished to obtain feedback about their lifestyles. Although all 10 (100%) participants actively followed their blood glucose levels, most of them (8/10, 80%) would have liked to receive more feedback on how to influence blood glucose levels.

Well, I would like to follow my general vitality, activity, sleep…like how stressed you are, do you eat well or not, are you able to keep a diary, so that your general wellbeing would be followed, not just the glucose slavishly but how to influence it.

Participant 8 (P8)

As the feedback they received was mainly negative, such as whether blood glucose levels are too high, 7 (70%) participants wished to also obtain positive feedback beyond physical activity

Well, the Garmin Vivosmart gives you feedback that you have achieved a goal, so a similar thing would be useful, that you would receive some kind of badge that everything has gone well.

P1

These results indicate that there is a lot of room for improvement in the amount and quality of the feedback to support self-discovery and further healthy behavior.

Many of the participants (7/10, 70%) wished to obtain clear suggestions on what to eat.

I wish it would give precise food suggestions, like what would be a good evening snack if I am having troubles with my fasting glucose in the mornings…so it would follow my diet and give me transformative suggestions so that the glucose values would stay within the limits so that it would be able to tell something like, now eat more protein but nothing else, or something like that.

P9

So, in addition to Harrison et al [37], who argued that women with GDM wish to obtain clear suggestions for physical activity, women with GDM wish for suggestions on nutrition as well. Ideally, these suggestions would be personalized according to the user’s diet.

I would like to have more tailored food suggestions, for example, if you are vegan…you could suggest replacing the oat milk with something else or I do not know…I miss out at least a half of these nutrition guides, as they are like, “Eat a rye bread sandwich,” but I can’t because I can’t eat rye bread.

P1

However, these suggestions considered not only the contents of the food but also the timing when one should eat. One participant implemented this by herself using alarms on her mobile phone.

I have had alarms “Remember to eat” on my mobile phone every two to three hours.

P10

In addition, 3 (30%) participants said that the suggestions should be provided gently.

For most of the participants, those tips must be useful. So if you replace that one with that one…at least I would be ready to receive that kind of tips, but I would not like to get a sermon; that does not help anything.

P8

Most of the participants (8/10, 80%) discussed that it would be motivating if they were able to find positive causes and effects of their behavior and blood glucose levels.

I think it would be insanely motivating if I were able to see that my glucose would stay within limits because I went for a walk.

P1

However, personalized recommendations do not currently exist in GDM apps.

Now you can’t get recommendations that walk for 45 mins and your blood sugar would go down. That would very valuable.

P3

Most of the participants (8/10, 80%) also requested precise and clear suggestions on lifestyle choices that they can control.

But could there be a feature in the app that would really say, you should eat in half an hour. Because that would be really good. And if my observation holds that even a small amount of physical activity decreases or balances blood sugar so it would be good if it said that “go for a walk.”

P3

This feedback should be provided gently and should respect the autonomy of women with GDM.

Here is this kind of recommendation that you would recover better by having longer sleep, having this kind of concrete suggestion when you are feeling tired with data that you slept badly would help. And the suggestion is not provided as an order that goes to sleep immediately…so it is given in a pretty pleasant form.

P6

The self-discovery process of finding how lifestyle influences blood glucose levels is tedious [8]. The app could facilitate this process by providing feedback and suggestions on what the person could try.

It would be good if you could get this kind of conclusion on what to try next, because now when you are figuring it out yourself, it is really like hit-and-miss what to try next…was it the sleep, or two pieces of bread or egg. So if it [the app] could help in this, it would be really great.

P9

Although our approach to providing recommendations was appreciated by 8 (80%) participants, providing feedback about sleeping, in particular, was not found to be useful.

I have only three and half weeks until the due date. I have no control oversleeping, for example.

P1

Thus, the feedback should be directed toward lifestyle changes that can be influenced by women with GDM.

To conclude, feedback (also positive) about lifestyle was highly appreciated, but it should be clear, feasible, and gentle. Feedback should also be directed toward lifestyle changes that women with GDM can influence, especially toward diet and physical activity by encouraging women to try different options for changing their diet and increasing physical activity. Suggestions for sleeping habits were not perceived as useful.

### Increase Autonomy by Enabling Personalization

Most of the participants’ (8/10, 80%) responses supported the importance of personalization.

Otherwise this looks good, but it would be good if you could modify this more based on one’s own needs so that if someone has problems with sugar levels and someone with physical activity…so everyone would be not be cut from the same cloth, and it could be slightly adjusted based on what one is able and wants to do.

P7

Personalization of habits was found to be motivating by over half (6/10, 60%) of the participants.

It would be motivating if you were able to add your own daily tasks, that I prefer more.

P3

Moreover, further evidence for the need for personalization of habits was revealed, as 3 (30%) participants found the proposed habits annoying.

I have to say about that “Maintain a hobby.” Being at this last stage [of pregnancy] is very laborious, and you know what kind of lifestyle you should have, but I don’t have enough strength really…I can’t have a hobby, so I am really annoyed by this kind of thing.

P3

Beyond our implementation, 7 (70%) participants discussed the personalization of goals for lifestyle choices. For example, the goal for physical activity should be customized to be achievable.

If I would realize at first that I am able to walk only 2000 steps a day, the 10 000 steps are pretty far. So if you could adjust the goal to be like 5000.

P3

Personalization was discussed for nutrition, as participants indicated that customized nutrition suggestions are important (see the Increase Competence to Manage GDM With Automatic Feedback and Interactive Exploration section).

The possibility to add a name, profile picture, and expected date of birth was not extensively discussed, although 3 (30%) participants preferred personal greetings as they made them feel nice.

Somehow this “Good morning, Suvi” feels nice, it feels personal…this is like a modern diary...so I would personally like to use this for sure.

P4

To conclude, participant responses reflected the relationship between personalization and autonomy. The ability of women with GDM to follow health recommendations, especially in terms of physical activity and sleeping, varies significantly between individuals and stages of pregnancy. Thus, these recommendations, user profiles, habits, and goals should be highly customizable. As these personalized recommendations are obtained, at least different options (eg, how to increase physical activity and what to eat) should be provided.

### Provide Social Support, Especially From the Partner

Incorporating this desirable feature raised divided opinions from participants. We observed that participants without previous children liked the functionality.

That walking together is very nice…maybe that is probably my favorite.

P4

However, participants who had given birth before were skeptical about this feature.

I think that in our case the partner’s/caregiver’s part in the app wouldn’t be used. Maybe for people who are pregnant for the first time, for whom all this is very new…so, wouldn’t be our thing, but [a] nice feature in general.

P8

Although the majority of the participants (6/10, 60%) preferred that their partners had a version of the app, the support needed from the partner varied. Some participants (4/10, 40%) discussed the role of the partner as providing support, such as a reminder to go sleep earlier or to provide encouragement in eating.

So it [the app] would notify my partner like [participant’s name] has slept badly, so ask her to go to bed earlier…that kind of additional feature would be good.

P4

It is good that the partner can see the weeks of gestation, and when thinking of gestational diabetes. It could remind my partner to encourage me in eating or something like that.

P5

As such, some of the reminders could originate from the partner, which could be more effective than providing reminders only for women with GDM. However, further studies are needed to investigate how women with GDM would perceive this.

Well, this [gestational diabetes] is also a bit of partner’s thing, or at least he gets influenced when I am saying aloud that my blood sugar is there so what should I do…my partner has also tried being on this same diet. I don’t know if it becomes oppressive if he monitors me; I do not know. But I think it would be nice if he could suggest today this and that from there [the app].

P9

In addition, 3 (30%) participants discussed that their partners would be willing to help if the reasons were clear.

At this stage, when the state of health is not the best, he [partner] starts to understand the realities, that something needs to be done and learned. So, I am sure he would receive the instructions and would be able to act accordingly if the reasons were justified well.

P10

However, 1 (10%) participant who did not find the partner’s view in the app useful stated that the app could be used to prove the changed conditions due to pregnancy.

You could use this as a weapon at home: “Look how little I have slept, so I need to sleep longer in the morning.”

P8

We also evaluated the femininity of the prototype app, as described in the Recruitment and Data Collection section. Although the participants rated the app’s femininity to be rather high (median 7), the ratings on femininity varied considerably (minimum=3, maximum=8). The ratings were more influenced by the topics related to pregnancy

Well, there is a picture of a baby, so it is probably the only feminine thing here.

P5

rather than the feel of the user interface:

There are nothing like stereotypical things, like flowers, hearts, and butterflies, which I have seen in period apps.

P6

The neutral and somewhat clinical look was preferred by the participants.

The feeling of medical science, makes it feel credible...if it was fluffy pink, I would not use it myself.

P1

The use of neutral elements and colors would support the usage of the app by both women with GDM and partners.

The community challenge raised fewer opinions. Of the 10 participants, 2 (20%) discussed belonging to a peer support group on social media and 1 (10%) participant found that this support could be improved by having things that women with GDM could do together.

I am part of a peer support group in Facebook, I have not been very active there, but people think about these things [issues about GDM] a lot and some things are left on their own, so it could be interesting if something like this is supported, at least like in this way virtually.

P9

However, most participants (8/10, 80%) did not comment on this feature.

To conclude, incorporating social support into the app divided opinions, and participants suggested different supportive roles for partners. The partner’s role in the app should be optional, and the partner should have their own version of the app. This partner’s version could include encouragement messages and reminders for a healthier lifestyle, which the partner can communicate with the woman with GDM. Some participants wished to share information about GDM with their partners so that the partners could support and help them in the management of GDM. The more advanced features, such as sharing data and suggestions for common activities, should be customizable.

### Support a Normal Pregnancy and Debunk Myths About GDM

Most of the participants (8/10, 80%) preferred having pregnancy information in the GDM app.

It is always nice to read what is the situation of the baby.

P3

I think that one can never have too much information about these things.

P4

I have this pregnancy app on my phone...I have looked at it almost twice a day from the beginning…this is something that I am interested in…how much it weighs and what parts have developed this time…so super big plus having this.

P8

This could be developed further by providing information not only about pregnancy but also about nutrition and physical activity.

Here is information about physical activity and pregnancy, [a] nutrition guide, information about gestational information…this is interesting, so it is more information than what I have seen from apps that I am using.

P6

To conclude, the participants valued having pregnancy and GDM information in the app. In addition, they wanted more information about nutrition and physical activity than what is available in current pregnancy apps. Optimally, this information should be personalized together with lifestyle suggestions (see the Increase Autonomy by Enabling Personalization section).

### Support Dual Processing as Pregnancy Is Life Changing

In general, almost all the participants (9/10, 90%) found our approach useful for supporting comprehensive self-tracking and to visualize these data in a single graph.

This seems somehow useful, as it is not only my own inference between two different graphs, so if I move then I would see how the blood sugar looks like, so having these graphs together seems like a good idea.

P6

Things that I like to follow: blood sugar, sleep, activity, those all are nicely and clearly shown together.

P2

Participants also embraced the possibility to select what factors to view in the visualization.

I like that you can choose in the graph which ones are visible, then you can compare blood sugar to activity or sleep or something similar.

P6

However, some participants (4/10, 40%) discussed that the possibility to select the factor did not simplify the view enough, as they thought that there was too much information and the view was too complex (eg, blood glucose information should be more distinct).

I think this [blood glucose] should be emphasized, visually it looks more like a background…so this glucose measurement is the most important thing, and these others are additional information.

P1

In addition, 1 (10%) participant requested highlighting blood glucose and having other data as an additional option.

In the graphs, there was too much information at a first glance. So, you could simplify this…well you can select to view the data that the user is interested in…so viewing all the graphs at the same time could be one possibility but maybe not the default option.

P5

Despite these concerns, the participants indicated that they would have benefited from such visualization at the beginning when they received the GDM diagnosis.

Concerning habitual visualization, 9 (90%) participants liked to have the glanceable data on the first page of the app. The importance of providing an arrow with blood glucose for indicating the trend (see Figure 1a) was also identified here.

I think those are great, like the glucose, there is an arrow, so it gives more information for what to compare.

P5

Some participants (4/10, 40%) indicated that they should pay attention to the app only when something is going wrong and thus support more reactive decision-making.

I think that the most important thing in the app would be like “Mom, relax. It will alarm when you need to do something about it.”

P1

Reminders were also discussed to help create measurement habits.

When I am having a post-meal measurement, I always add it as an alarm to my mobile phone. So, of course, if that would be possible using the app, I would not have to do it separately.

P3

It could remind me when I need to measure more than the normal blood sugar in the morning.

P8

The mobile phone was mentioned as being useful for tracking habits, as people follow their mobile phones anyway.

For example, the goals, like habits and so on, what you can put there…that feels something that would be useful for me. So when you add them and the app would remind me, it would be something that I would follow, because I follow the mobile phone quite often. If you had a separate diary or something like a paper or calendar, then you would not most likely notice them.

P6

As such, in addition to customizing the habit (see the Increase Autonomy by Enabling Personalization section), the reminders of these habits could also be customized. A participant discussed that once healthier habits have become part of everyday life, assistance from the app would not be needed anymore.

You would need to follow the app every day pretty slavishly…so I think one of the goals of this app would be to learn to be without it…the less you would need it, the better. Now it is quite engaging…so there should be a balance…the app is more like help but not something that flogs.

P7

To conclude, both approaches (habitual and reflective) to support decision-making should be incorporated in the design. The dual processing responds to the various needs of women with GDM, as some were interested in investigating the self-tracking data in depth, whereas others preferred the glanceable information about current values. It is recommended to use simple visualizations (eg, using the traffic light metaphor or arrows) to show real-time feedback and line charts combining blood glucose levels and lifestyle for providing detailed information about the causes and effects. Participants had different opinions on how much they are willing to pay attention to data and how much should be provided in the form of notifications and alarms.

### Integrate the App With Normal Pregnancy and Existing Health Care Services

In addition to supporting desirable features, providing information that is common for pregnant women in general and women with GDM supported the feeling of normal pregnancy. Having the gestational weeks on the first page was clear for the participants.

Pregnancy weeks are easy to understand.

P1

The overall appearance of the app looks clear as there are pregnancy weeks on the first page.

P7

Providing pregnancy information in addition to self-tracking data was appreciated by the participants (8/10, 80%).

It’s nice that you can go from the first page to pregnancy information, so everything is not just hard data, but also information similar to pregnancy applications.

P10

Almost all the participants (9/10, 90%) suggested that the app could be part of health care, especially if sufficient instruction is provided.

I think this should be some kind of maternity clinic app right away, and good instruction should be provided.

P1

A pretty good introduction should be given at the start, and then there could be a possibility to quit using it [the app] if it doesn’t feel one’s thing. If you are new and suddenly you are given this kind of app, it can be that it doesn’t suit everyone.

P7

Participants discussed the importance of having contact with a diabetes nurse so that they can share the data with them and discuss the data provided by the glucose sensor.

I think that a chat and feedback functionalities would be useful, so it would send an alarm if you get a lot of hypers, and that information should be automatically transmitted to the diabetes nurse…I think the current way is very old school…now we send some glucose values by email, which can basically be anything as they can vary from time to time.

P1

Many of the participants (7/10, 70%) wanted to receive a message from the app to contact a clinic if something unusual occurs.

There should be contact details for maternity clinics or something like that. So, when you face some challenges, for example, if you have had three hypers this week, you would get a notification to contact the clinic.

P4

More than half of the participants (6/10, 60%) also mentioned that the app should be integrated with visits to a clinic and the app could support collecting more data and provide them for health care personnel.

You could utilize the app in maternity clinics so that there weren’t so many different units…that you could combine this to maternity clinics, so this could be a good package for them. Then you could also add blood pressure or weight or something to the app you once have visited the clinic.

P7

To conclude, participants liked to have gestational weeks on the first page and pregnancy information to support the feeling of normal pregnancy. They also felt that the app could be part of their routine care and provided different suggestions on how the app could be integrated into health care services, including good instructions, use of self-tracking data for visits to maternity clinics, and enabling connection to the maternity clinic when blood glucose levels have frequently been exceeded.

### Other Emerged Themes

Most of the comments from women with GDM were consistent with literature-based desirable features [14], indicating that desirable features cover the majority of themes discussed in the interviews. However, as we analyzed emerging themes beyond the literature-based desirable features, we want to note the following important aspects.

#### Basic Functionalities That Are Simple to Learn but Have Sufficient Depth to Maintain Interest

The temporal dimension (ie, how the use of the app would vary as a function of time) was not extensively discussed in the desirable features. However, this issue was raised in the interviews by 4 (40%) participants.

The more I would use this app, the more I would benefit from it…the first week I would be filling information, but then I would start setting habits and other things and would gain more benefits.

P8

This emphasizes the need for good instructions when using the app, which was discussed in the Integrate the App with Normal Pregnancy and Existing Healthcare Services section. After the initial stage, the app should have sufficient depth for maintaining interest.

There should be more features rather than less, so it is versatile. And it [the app] is a sort of combination of things that I would use anyway, so having a lot of features doesn’t bother me…it maintains interest if there are many different things.

P4

As such, provided that the usability is not compromised due to multiple features, having them would be beneficial. This seemed to be the case for our app.

This [app] is pretty clear and simple…and after using it a couple of minutes this feels easy to learn and grasp…when compared to other apps I’m using and have used.

P10

#### Tolerance for Slipping and Usage Breaks

Half of the participants (5/10, 50%) discussed that the app should be started on the mother’s own terms (see also the Increase Competence to Manage GDM With Automatic Feedback and Interactive Exploration section regarding starting self-tracking). This includes tolerance for slipping, which happens to women with GDM as well.

I would consider this app interesting as I slip sometimes…but it [the app] should not increase anxiety if these slips are recorded there…No one is perfect, and there could be some things that you at least won’t be blamed for if you slip…If you need to show this to someone, you might neglect some markings or not use it if you feel ashamed that you haven’t been that good. So, having that kind of app would be the best option.

P7

This emphasizes that the app should focus on the positive aspects of how person is managing GDM, for example, through positive feedback (see the Increase Competence to Manage GDM With Automatic Feedback and Interactive Exploration section). In addition, 3 (30%) participants discussed that their attitude toward the app might vary time to time.

For most of the time, the app would be like a good thing, but I can imagine that there would be times that the whole app would start annoying me.

P2

This indicates that the app should be tolerant of usage breaks and different usage frequencies.

## Discussion

### Principal Findings

In this study, we examined how to incorporate literature-based desirable features [14] into mobile apps designed for management of GDM. Most of the feedback on example implementations of the desirable features was positive and constructive. We identified that self-tracking data in GDM apps should be extended with written feedback, habits and goals should be highly customizable to be useful, providing social support through the app should be possible for the partner, and health care professionals should be notified through the app if something unusual occurs. Relating to automatic feedback and self-tracking data exploration (desirable feature 1), the feedback through the self-tracking data was of value, but the interviews revealed that the feedback should be extended with written feedback. The interviews also indicated that the guidance should be clear, feasible, and gentle. In general, these comments are consistent with findings by Harrison et al [37], which indicated that women with GDM want clear suggestions on what physical activity to engage in. However, this should be provided gently and should consider personal physical capabilities and preferences for diet, respecting the women’s autonomy. Many women encounter difficulties in trying to adapt ethnic diets to the dietary changes that GDM demands [27,38,39]. Draffin et al [27] considered giving culturally appropriate advice as crucial to prevent women from feeling alienated, as it can be difficult for women to follow recommendations when they feel that such recommendations are not applicable to their lives. Providing automatic guidance in the form of recommendations or other written feedback is largely missing in existing GDM apps [14]. Thus, future work is needed on how to provide influential feedback for women with GDM with automatic approaches to achieve the effectiveness of human feedback [14].

Regarding the personalization possibilities in the app (desirable feature 2), our results support findings by Goetz et al [40], who reported that personalized welcome messages and the possibility to add their pictures were appreciated by a third of pregnant women. However, participants stressed the importance of personalizing daily habits and goals, which has not been possible in existing GDM apps [41]. As such, there is a lot of room to enhance the personalization features of GDM apps. Personalization should not be limited to lifestyle choices but should also include personal concerns related to pregnancy [38]. How to personalize apps for GDM for women from different ethnic and cultural backgrounds in managing their diabetes requires further studies.

Social support (desirable feature 3) has also been absent, as none of the existing GDM apps provides social support from the partner [14]. The responses from participants indicated that their partners could have different supportive roles through the app. More investigations are required to determine how this could be implemented such that women with GDM do not feel controlled but are supported in making healthier lifestyle decisions. Showing simple notifications to the partner, without accessing the woman’s self-tracking data, would be a good starting point as the amount of data disclosed to the partner would be minimized. The user could then decide whether they would be willing to share more data with their partner. In addition, the partners’ opinions about the intended functionalities for them should be studied more. These investigations have been conducted for pregnancy apps designed for men [42] but are lacking in GDM apps [14]. Beyond partners, providing social support through a community challenge did not raise many comments from the partners. This is consistent with the findings by Peyton et al [26], who showed that more research is needed on how to incorporate social media activities in pregnancy apps.

Regarding debunking myths about pregnancy (desirable feature 4), the responses were consistent with the literature that women with GDM want to have GDM and pregnancy information in the same place [17]. However, participants requested more in-depth information about nutrition and physical activity than the current apps provide, akin to a previous study [17]. As such, GDM apps should have a comprehensive information section about pregnancy, GDM, and related topics, such as nutrition (eg, recipes) and physical activity.

Regarding the depth of information, the participants’ willingness to interpret the self-tracking data significantly varied. Some wanted to discover cause and effect, whereas others just wanted to know “if something is wrong.” This indicates that support for dual processing (desirable feature 5) is useful. The dual-process approach is supported fairly well in current GDM apps in terms of providing data at varying levels of detail [14]. Habitual processing is implemented with glanceable visualization of recent blood glucose levels, whereas more detailed graphical visualization is provided to support reflective processing. Habitual or reactive processing is further supported with reminders and alarms. However, the possibility to create and track daily habits is missing from current GDM apps [14]. Participants would especially appreciate habit tracking if they were able to personalize their habits (see desirable feature 3). Moreover, linking the new or changeable habit to an existing habit can be expected to be a more effective approach than time- and phone-based approaches in the long term. How this could be accomplished remains an open challenge, but as the techniques for activity recognition continue to develop [43], detecting the existing habits may soon become possible, thus enabling linkage of new and changeable habits with existing habits, such as measuring blood glucose levels, which is a frequent task for women with GDM.

Finally, regarding desirable feature 6, integration to a normal pregnancy was appreciated as participants discussed having gestational weeks on the first page as a good and familiar feature from pregnancy apps. Participants also proposed ways for how the app could be integrated into health care services in terms of monitoring between maternity clinic visits. These findings are in line with the review on technological support for diabetes management, which emphasizes the importance of 2-way communication between people with diabetes and health professionals [29] and that the self-tracking with wearable sensors can increase the completeness of the self-tracking data presented to health care professionals [44]. However, it is unclear how health care professionals would perceive self-tracking data beyond blood glucose levels. A big issue on acceptability is their attitude toward the data provided by lifestyle and well-being devices [44,45]. These acceptability investigations of well-being data among health care professionals are missing in the context of GDM. This integration has been partly implemented in current GDM apps [46,47], and the absence of this integration is considered a large shortcoming [17].

In addition, we found 2 important themes from the interviews that were not clearly related to desirable features. First, as GDM is perceived as “a stun” [48], the participants indicated that the app should be simple to take into use. This is particularly important at the beginning, when some effort is required for setting up the app. There should be also deeper features to maintain interest for women with GDM at a later stage of pregnancy. We believe that it is possible to design an app that is easy to use but has many features. This is in contrast to the findings by Hood et al [49], who observed that the number of features decreases the usability of diabetes apps in general. However, keeping the basic functionalities simple and having sufficient depth from additional features seem to be a good approach. Second, as women with GDM may have feelings of guilt, the app should have a tolerance and supporting approach for slipping and unfavorable behavior. Skar et al [17] discuss that slipping might lead to undesirable “cheating.” This also implies that returning to the app after a while should be easy and that lack of use should not be punished but rather supported by friendly messages to guide women with GDM to start using the app again.

### Limitations of the Study

We are aware that the design space for implementing each desirable feature is large, and thus, multiple different implementations can be derived from it. At this stage, we used these example implementations of desirable features as starting points to explore and evoke further ideas from participants. As such, our aim was not to validate these implementations of the desirable features but to gain constructive feedback to incorporate the desirable features into a future GDM app.

We acknowledge that the number of participants could have been higher. However, we believe that this was sufficient as the data seemed to be saturated after 8 interviews. Moreover, the same number of participants have been used in qualitative studies on experiences of GDM (eg, [48]).

The participants were familiar with using mobile apps, which reflects the wide adoption of mobile apps among pregnant women in developed countries [50]. However, more work is needed to assess how acceptable and feasible the app-based interventions are for women with GDM in large-scale use [18].

### Directions for Future Work

Most of the studies on GDM apps have investigated the effects of these apps on medical outcomes or user experience. However, less work has focused on constructive approaches for investigating the design features of GDM apps. This study aimed to fill the gap by designing and providing examples of features that have justifications in the literature [14] and by providing the perspectives of women with GDM. In the future, these features should be implemented and integrated into a functional GDM app to investigate their impact on GDM self-management. More research is required to understand the efficacy of each exploratory desirable feature provided here. To do this, we will incorporate at least part of these features in a fully functional mobile app for iOS and Android and conduct a randomized controlled trial to evaluate its effect on maternal and neonatal outcomes in the future. In addition, the feasibility of implementing each desirable feature is largely unknown. However, the implementation of providing reliable information can be considered to be much easier than the implementation of habit formation and tracking, for example.

An interesting part of future work is to study interactive learning environments with women with GDM that allow creating causalities between health behaviors (diet, physical activity, and sleep) and blood glucose levels. Especially, this would support desirable features 1 and 4. A survey on the features of diabetes apps by Adu et al [51] revealed that the teaching of diabetes self-management skills is underrepresented in diabetes apps ﻿[52], although ﻿recent guidelines emphasize the need for patient education [53]. Qualitative studies [21,27] indicate that the self-discovery process of GDM is challenging and demanding, which takes a considerable amount of time. Nevertheless, the self-discovery process could be expedited and facilitated. For example, this could be achieved with interactive visualizations of how lifestyle impacts blood glucose levels and what kind of potential impact this can have on the child. Bień et al [54] noted that increased knowledge of diabetes during pregnancy increases perceived general health. Carolan-Olah et al [55] suggested that knowledge of GDM could be improved, particularly for women from multiethnic and low socioeconomic backgrounds. Although an app is a common way to provide an eHealth intervention, the interactive learning environment does not necessarily need to be restricted to apps. An interactive learning environment could be leveraged to other technologies for diabetes management, as explored by Rollo et al [56]. These include web-based programs, video games, and even emerging technologies, such as virtual reality and augmented reality. For example, the use of virtual reality can provide an immersive environment to rehearse healthier choices [56]. However, it is still unclear what benefits these technologies could bring specifically to GDM management over mobile health (mHealth) learning environments.

### Conclusion

This study was intended as a constructive step in the design and development of a mobile app to support behavior change required in the self-management of GDM. We designed and evaluated how to incorporate desirable features into GDM apps. The results confirmed the importance of these features to support self-management through GDM apps, and we gained constructive information about the contents and functionalities of each feature. We expect that the implementation of at least some of these features will increase the efficacy of GDM self-management apps. We argue that the results are useful for various stakeholders that target effective mHealth interventions for GDM.

## Abbreviations

GDM: gestational diabetes mellitus
mHealth: mobile health
